# Intra-muscular follicular dendritic cell sarcoma in the thigh: A case report

**DOI:** 10.1097/MD.0000000000027209

**Published:** 2021-09-10

**Authors:** Yong Jin Cho, Song Iy Han, Sung-Chul Lim

**Affiliations:** a Department of Orthopedic Surgery, College of Medicine, Chosun University, Gwangju, Republic of Korea; b Division of Premedical Science, College of Medicine, Chosun University, Gwangju, Republic of Korea; c Department of Pathology, College of Medicine, Chosun University, Gwangju, Republic of Korea.

**Keywords:** extranodal, follicular dendritic cell sarcoma, intra-muscular, soft tissue, thigh

## Abstract

**Rationale:**

Follicular dendritic cell sarcoma (FDCS) is an intermediate-grade malignancy originating from follicular dendritic cells. Nodal FDCS is the most common type, meaning that the extranodal type may not be recognized and could be easily misdiagnosed. Reported extranodal sites include the head and neck, retroperitoneum, spleen, liver, and gastrointestinal tract. FDCS in the soft tissue is extremely rare.

**Patient concerns:**

A 75-year-old male presented with complaints of a localized swelling and intra-muscular soft tissue mass in the left upper thigh.

**Diagnosis:**

The present tumor consisted of fascicular or vague storiform-arranged spindle cells with less pleomorphism and many lymphoid aggregates. Tumor cells were positive for CD21, CD35, CD68, vimentin, and EGFR. Intra-muscular FDCS was confirmed by immunohistochemical studies.

**Interventions:**

The patient received a wide marginal excision, followed by adjuvant radiotherapy.

**Outcomes:**

Symptomatic improvements were achieved and no subsequent relapses were observed.

**Lessons:**

If the tumor arises in the extranodal sites, especially in the soft tissue, it is difficult to include FDCS in the differential diagnosis. When the immunoprofile is not consistent with that of common spindle cell tumors, immunostaining for follicular dendritic cell markers such as CD21, CD23, and CD35, as well as further immunohistochemistry for D2-40, CD68, EGFR, Epstein-Barr virus, and BRAF can be helpful for the diagnosis and subtyping of FDCS. To the best of our knowledge, the present case is the first case of intramuscular FDCS.

## 1 Introduction

Follicular dendritic cell sarcoma (FDCS) is an extremely rare intermediate-grade malignancy originating from follicular dendritic cells (FDCs), which are essential for the formation of lymphoid follicles comprising B cells. Accordingly, FDCS involves lymph nodes and constitutive lymphoid tissue, as well as acquired lymphoid tissue. Although most lymphoid-associated dendritic cells are of myeloid origin, FDCs are regarded to be of mesenchymal origin.^[1,2]^ Since its discovery in 1986, FDCS has been reported to generally occur in lymph nodes, with less than one-third cases of FDCS having been described in extranodal sites.^[3,4]^ The most common extranodal sites are the intra-abdominal organs, followed by the head and neck; however, involvement of the thorax, thyroid, skin, and breast has also been reported. Although FDCS usually exhibits indolent behavior, intra-abdominal cases show more aggressive clinical behavior.^[1,5,6]^ As an extremely rare tumor, FDCS occurring in extranodal sites has occasionally been misdiagnosed, as it is rarely considered in the differential diagnosis. FDCS occurs in all age groups, but is most frequently observed in young to middle-aged adults, with no sex predilection.^[1,7]^

In the English literature, 1 case of FDCS has been reported in the extremities, with a tumor found in the thigh of a 6-year-old female. Although further detailed information on clinical presentation, including the specific location within the thigh, was missing, considering the histologic pattern described as “interlacing fascicles of spindle cells with sprinkling of lymphocytes” and the referring pathologist's diagnosis reported as spindle cell sarcoma, this case seems to be classic type FDCS.^[6]^

Here, we report the case of a tumor consisting of vague storiform and fascicular arrangement of plump spindle cells, presenting as an intra- and inter-muscular lesion of the thigh, which was diagnosed as FDCS based on the positive immunoreactivity for the FDC markers CD21 and CD35. In addition, mature lymphocytes sprinkled throughout the tumor or forming dispersed aggregates were reminiscent of inflammatory pseudotumor (IPT), leading to the differential diagnosis of IPT-like FDCS. In light of its rarity and unusual clinical presentation, we report the first case of FDCS presenting as an intra-muscular lesion in the extremity, in addition to a review of the relevant literature.

## 2 Case report

This study was approved by the institutional review board of the Chosun University Hospital (Permission number: CHOSUN 2021-02-006). Informed written consents were obtained from the patients for publication of this case report and accompanying images.

A 75-year-old male presented with complaints of a localized swelling and mass in the left upper thigh. Although the lesion had been recognized about 11 months prior, treatment was not administered until the patient visited a local clinic 3 months ago, owing to gradual growth of the lesion. A 5 × 5 cm mass was detected upon ultrasonography, and the patient was subsequently advised to visit our hospital. No history of trauma or inflammation was reported, and the patient complained of no specific pain except discomfort when sitting down. Past medical history included hypertension for 15 years, diabetes for 20 years, and benign prostatic hyperplasia. The patient had received surgery for lumber vertebral disc herniation 12 years ago and total knee arthroplasty on both knee joints 6 years ago. No significant weight change, fever, or fatigue were noted.

**Figure 1:**
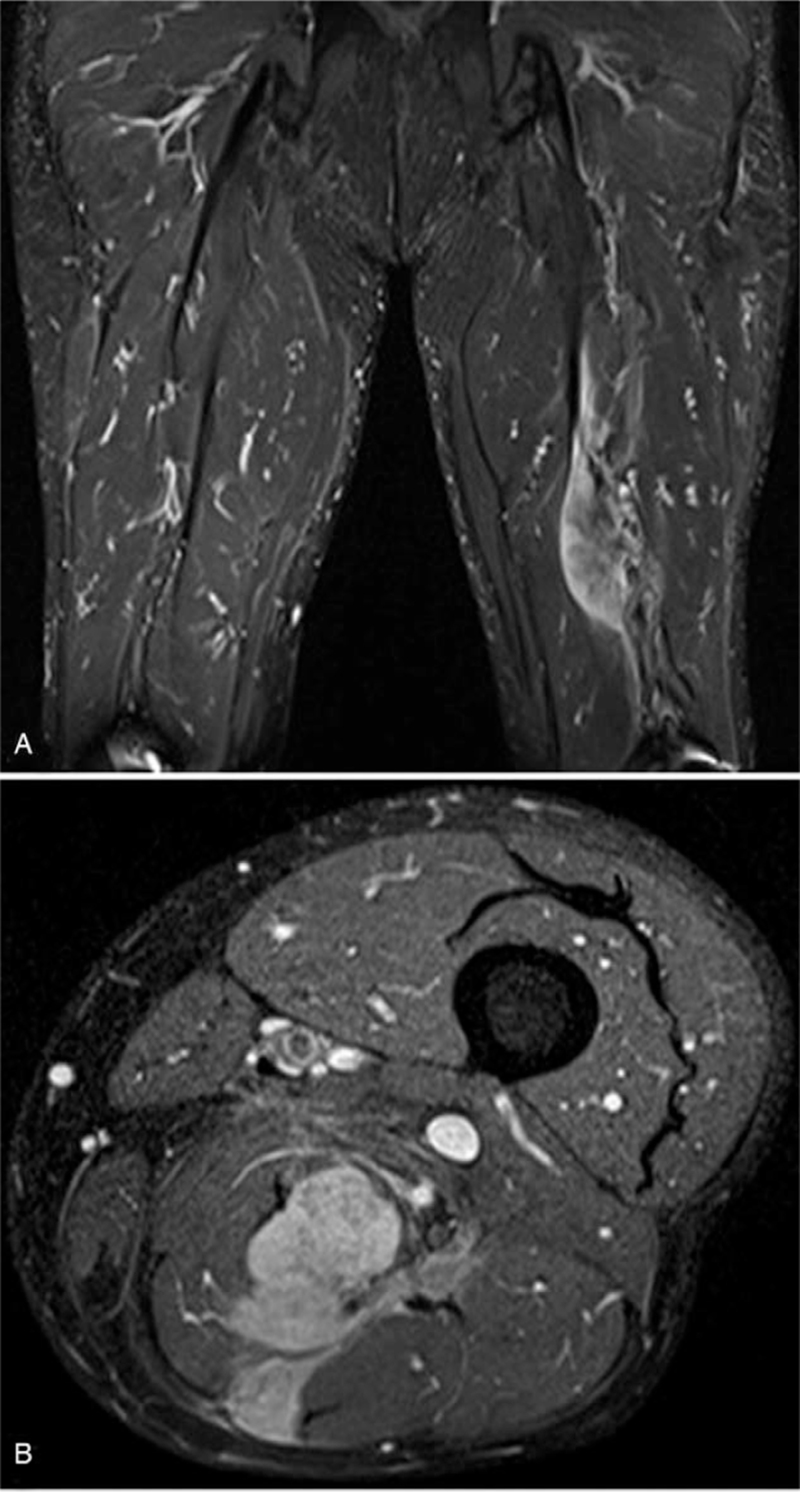
MRI showed a 12.5 × 6 × 3.5 cm sized irregular abnormal signal intensity with a vivid, enhancing soft tissue mass in the left posterior thigh, along the semimembranosus and semitendinosus muscle. MRI = magnetic resonance image.

Magnetic resonance images showed a 12.5 × 6 × 3.5 cm sized irregular abnormal signal intensity and vividly enhanced space indicating a soft tissue mass in the left posterior thigh, surrounded by the semimembranosus muscle and semitendinosus muscle. This inter- and intra-muscular lesion had invaded the superficial investing fascia and adjacent sciatic neurovascular bundle (Fig. 1). The clinical impression was soft tissue sarcoma, and a wide marginal excision was performed. After surgery, the size of the mass invading the adjacent skeletal muscle was found to be 4.5 × 4.5 cm; The larger size observed upon magnetic resonance image may be attributed to fibrosis and the change in muscle fibers induced by tumor invasion. To obtain safety margins, mass resection including the adjacent normal skeletal muscle was performed. However, since the tumor encircled the sciatic nerve and adhered to the nerve sheath, complete surgical resection was impossible, and a residual lesion was therefore left in the region abutting the sciatic nerve.

**Figure 2:**
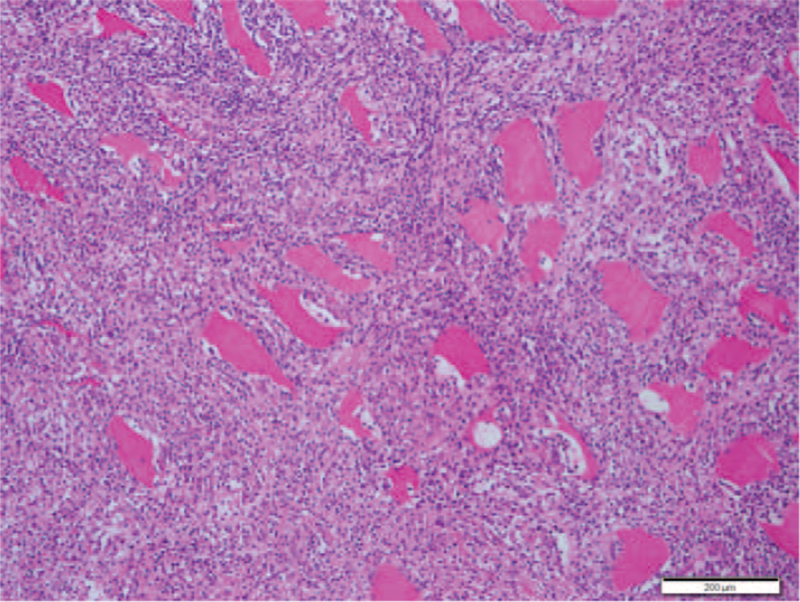
Histopathological findings of intra-muscular mass. It was found to be unencapsulated and showed an infiltrative growth into the adjacent skeletal muscle. Scale bar measures 200 μm.

**Figure 3:**
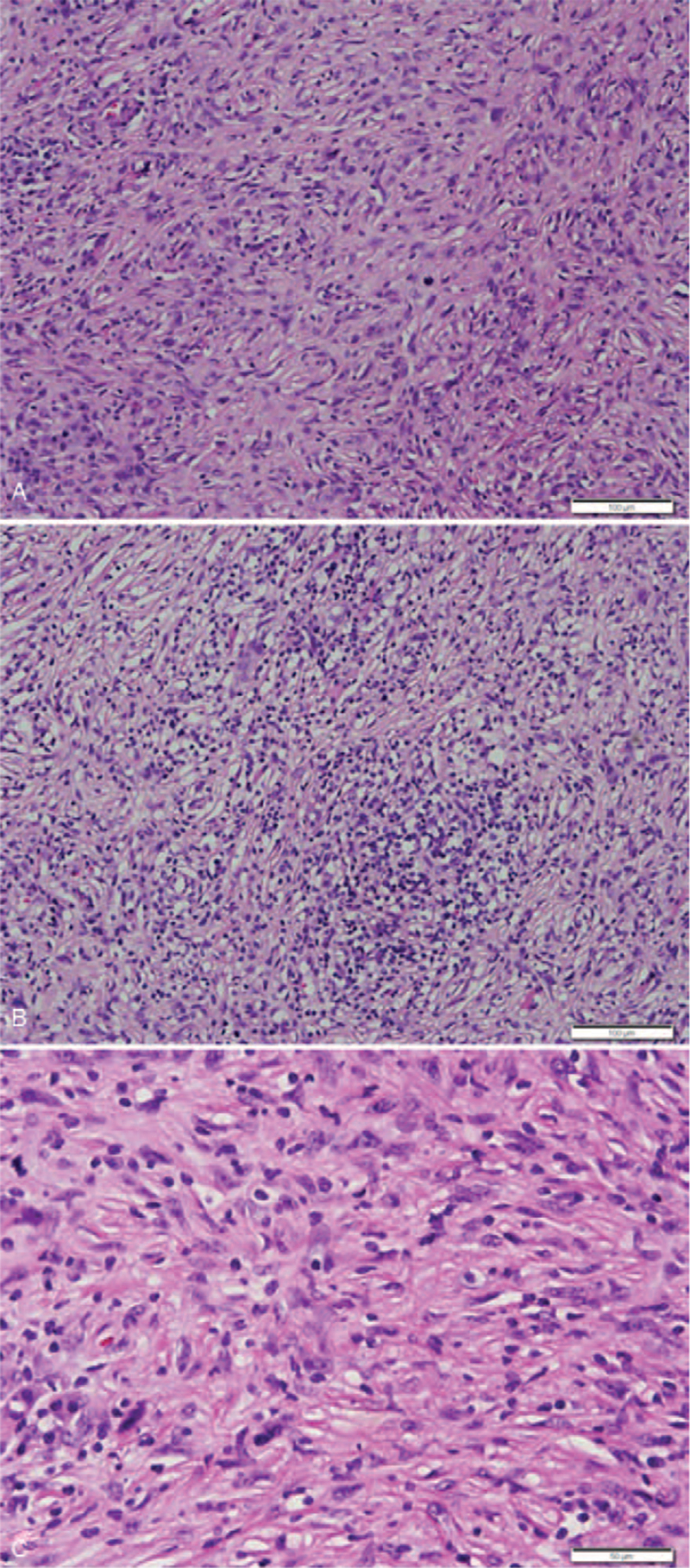
Histopathological findings of follicular dendritic cell sarcoma. (A) The tumor consisted of spindle cells forming short storiform and vague interlacing fascicles with sprinkled lymphocytes. (B) The tumor showed less cellularity with lymphoid aggregates. (C) A high-power view of the area in A showed spindle cells composed of elongated, vesicular, sometimes irregularly twisted, nuclei with small nucleoli and occasional atypical mitosis. Scale bars measure 100 μm (A, B) and 50 μm (C).

**Figure 4:**
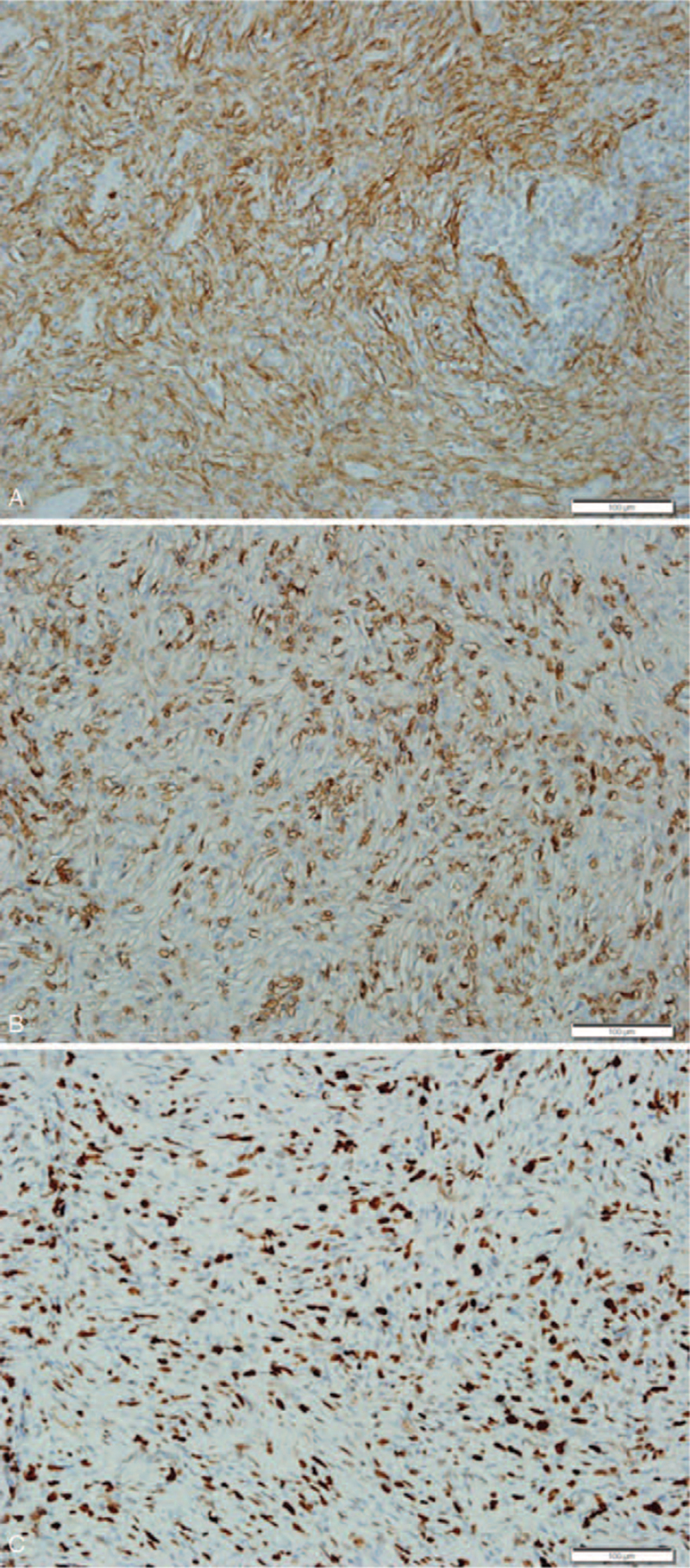
Immunohistochemical findings. (A) Spindle cells showed positivity for CD21, but not in the lymphoid aggregates. (B) Spindle cells showed positivity for CD35. (C) Ki-67 labelling index showed expression in 43% of the tumor cells. Scale bars indicate 100 μm.

Upon histopathologic examination, the mass was found to be unencapsulated and showed an infiltrative growth into the adjacent skeletal muscle (Fig. 2). The tumor was composed of an admixture of chronic inflammatory cells and spindle cells. The spindle cells were haphazardly distributed or arranged in vague fascicles or a storiform pattern. The chronic inflammatory cells mostly consisted of mature lymphocytes and plasma cells, and the main cellular component was spindle cells with sprinkled lymphocytes (Fig. 3A), with lymphocytes distributed in aggregates (Fig. 3B). This growth pattern was considered similar to that of IPT. At high power, the spindle cells showed an indistinct cell membrane and were found to be eosinophilic with slightly fibrillar cytoplasm. The nuclei were elongated and vesicular, and some were irregularly twisted. Although cellular pleomorphism was minimal, based on small nucleoli, cytologic atypia, and occasional atypical mitoses (averaged 2/10 high power fields), spindle cell sarcoma was considered (Fig. 3C). No necrosis was observed. Immunohistochemically, the spindle cells showed diffuse positivity for CD21, CD35, CD68, vimentin, and EGFR (Fig. 4 A, B), and focal positivity for D2-40 (podoplanin) and EMA. The spindle cells were negative for α-smooth muscle actin, ALK, CD23, CD34, BRAF, Epstein-Barr virus (EBV) Epstein-Barr virus-encoded small RNAs (EBER), and S-100 protein and had a Ki-67 labelling index of 43% (Fig. 4C). The background lymphocytes were mainly CD3-positive, sometimes CD20-positive, and EBV-negative. Presence of the BRAF V600E mutation was investigated via real-time polymerase chain reaction of the paraffin-embedded tissue block; however, no mutation was detected. In addition, the presence of mutations in EGFR on chromosome 7p11.2 was examined using polymerase chain reaction and pyrosequencing; no mutations were identified in exons 18, 19, 20, and 21. Taken together, the final diagnosis was extranodal FDCS, showing an extremely rare clinical presentation.

The patient received adjuvant radiotherapy at 3.5 weeks after surgery. The radiation dose was 180 to 200 cGy daily in 5 fractions/week, resulting in a total dose of 66 to 70 Gy/7 weeks. As of now, no signs of recurrence have been observed after 14 months of surgery.

## 3 Discussion

FDCS, the most common histologic subtype of dendritic cell tumors, is an intermediate-grade sarcoma with a propensity for local recurrence (28.1%) and distant metastasis (27.2%). Similar to that in other soft tissue sarcomas, it is known that tumor size of ≥ 6 cm, the presence of tumor necrosis, high mitotic count (≥5/10 high-power fields), and cytologic atypia are associated with poor prognosis,^[8,9]^ although 1 prior study reported that tumor necrosis has no association with prognosis.^[10]^

According to histological morphology, FDCS can be classified into the classic type and the IPT-like type. IPT-like FDCS is much rarer than classic FDCS and has distinctive features. IPT-like FDCS occurs mainly in the liver and spleen, has a marked female predilection (F:M = 2.2:1), and rarely recurs or metastasizes.^[7,11,12]^ Regarding the aspects of histological morphology and biological behavior, IPT-like FDCS is considered to be completely different from the classic type and is consistently associated with EBV, and present in monoclonal episomal state.^[7,11–13]^ Present case showed EBV negativity by EBER, so IPT-like FDCS could be ruled out. Moreover, the lymphoid infiltrate in this case did not appear heavy, only sprinkling with occasional aggregates. Such a lymphoplasmacytic infiltrate is typically seen in classic FDCS. No significant eosinophilic infiltrate is noted either. If the tumor has a heavy lymphoplasmacytic infiltrate with or without eosinophils, obscuring the neoplastic FDCs and really mimicking ‘inflammatory pseudotumor’, IPT-like FDCS could be considered as a differential diagnosis.

It is difficult to diagnose FDCS in cases where the tumor consists of bland spindle cells with no nuclear atypia admixed with chronic inflammatory infiltrates and is present in extranodal sites. In the present case, the tumor consisted of spindle cells, forming vague fascicles and storiform patterns, and lymphoplasmacytic cells, which were reminiscent of IPT, thus indicative of an inflammatory myofibroblastic tumor. However, the results of immunohistochemical staining showed α-smooth muscle actin negativity, based on which other tumors should be considered in the differential diagnosis. Despite minimal cellular pleomorphism, the cytologic atypia and occasional atypical mitoses (averaged 2/10 high-power fields) warranted the consideration of a malignant spindle cell tumor. Since additional immunohistochemical staining showed negativity for S-100 protein and positivity for vimentin, FDCS was considered despite its rarity. Based on the strong reactivity for CD21, CD35 and CD68, and EBV negativity by EBER in spindle cells, a diagnosis of FDCS was made. Likewise, discordant diagnoses are common in the initial evaluation of IPT-like FDCS, which have been misdiagnosed as Hodgkin lymphoma,^[14]^ malignant fibrous histiocytoma,^[15]^ and myofibroblastic tumor.^[11,16]^ Therefore, when an unusual immunoprofile is observed upon the immunohistochemical examination of a malignant spindle cell tumor, additional immunostaining for CD21, CD23, CD35 could be helpful to rule out FDCS, although this tumor is rare. In addition, 1 study reported that the spindle cells of FDCS show strong, diffuse immunoreactivity for D2-40, with a membranous and cytoplasmic staining pattern, suggesting D2-40 as a highly sensitive marker^[17]^; in the present case, D2-40 showed a focal positivity.

Although BRAF mutations can be found in both the classic and IPT-like types (18.5%), it was reported that the mutation is markedly observed in the IPT-like type (40%) and, accordingly, could be helpful to distinguish between the 2 types.^[18]^ BRAF mutation was not detected in the present case. It has been reported that the spindle cells in FDCS are positive for CD68^[8,19]^ and EGFR^[4,20]^ on immunohistochemical staining; additionally, the key role played by EGFR in the tumorigenesis of FDCS by driving the survival and proliferation of tumor cells has been previously verified.^[20]^ In the present case, immunohistochemical staining revealed positivity for CD68 and EGFR, although no *EGFR* mutation was found.

Extranodal FDCS is extremely rare and generally affects the intra-abdominal organs and the head and neck. In addition, there have been scattered reports on its occurrence in the retroperitoneum, paratracheal region, lung, mesentery, thyroid, parotid, paravertebral region, orbit, nasopharynx, and mediastinum. Moreover, FDCS occurs more rarely in the soft tissue, and only 1 case has been reported in the extremities, which affected the thigh of a 6-year-old girl.^[6]^ However, since the specific site was not described in this report, the present case seems to be the first intra-muscular FDCS reported in the English literature.

The established treatment protocol for patients with FDCS is not present due to the low incidence and the variable clinical courses. However, main treatment for FDCS is surgical resection in majority of early local cases. Chemotherapy and/or radiotherapy can be applied in advanced cases or incompletely resected tumors.

According to the research report,^[21]^ 11/23 (48%) patients underwent either adjuvant radiation (9/11, 82%) or neo-adjuvant chemotherapy with doxorubicin and ifosfamide-based regimens (2/11, 18%). The 5-year overall survival for patients who received adjuvant or neo-adjuvant therapies (n = 11) and those who were observed (n = 12) were 39% and 69%, respectively; however, this difference was not statistically significant (*P* = .58).

In the present case, since the tumor encircled the sciatic nerve and adhered to the nerve sheath, it was impractical to carry out complete removal, and further radiotherapy was performed. This tumor generally has a low recurrence rate and a favorable long-term outcome^[22]^; in line with this, the present case has shown no evidence of disease for 14 months after surgery.

## 4 Conclusion

To the best of our knowledge, only 1 case of FDCS in the thigh has been reported to date. The present case seems to be the first report of intra-muscular FDCS. Regardless of its subtypes, FDCS is an extremely rare tumor, with no characteristic clinical or imaging features. Thus, if the tumor arises in the extranodal sites, especially in the soft tissue, it is difficult to include FDCS in the differential diagnosis, potentially leading to misdiagnosis. When the immunoprofile is not consistent with that of common spindle cell tumors, immunostaining for FDC markers such as CD21, CD23, and CD35, as well as further immunohistochemistry for D2-40, CD68, EGFR, EBV, and BRAF can be helpful for the diagnosis and subtyping of FDCS.

## Author contributions

**Data curation:** Yong Jin Cho, Song-Iy Han.

**Funding acquisition:** Sung-Chul Lim.

**Methodology:** Yong Jin Cho, Song-Iy Han.

**Supervision:** Sung-Chul Lim.

**Validation:** Sung-Chul Lim.

**Writing – original draft:** Yong Jin Cho.

**Writing – review & editing:** Song-Iy Han, Sung-Chul Lim.

## References

[1] Biddle DA Ro JY Yoon GS. Extranodal follicular dendritic cell sarcoma of the head and neck region: three new cases, with a review of the literature. Mod Pathol 2002;15:50–8. 11796841 10.1038/modpathol.3880489

[2] Muñoz-Fernández R Blanco FJ Frecha C. Follicular dendritic cells are related to bone marrow stromal cell progenitors and to myofibroblasts. J Immunol 2006;177:28028–9. 10.4049/jimmunol.177.1.280 16785523

[3] Monda L Warnke R Rosai J. A primary lymph node malignancy with features suggestive of dendritic reticulum cell differentiation. A report of 4 cases. Am J Pathol 1986;122:562–72. 2420185 PMC1888214

[4] Wu A Pullarkat S. Follicular dendritic cell sarcoma. Arch Pathol Lab Med 2016;140:186–90. 26910224 10.5858/arpa.2014-0374-RS

[5] Youens KE Waugh MS. Extranodal follicular dendritic cell sarcoma. Arch Pathol Lab Med 2008;132:1683–7. 18834231 10.5858/2008-132-1683-EFDCS

[6] Kaur R Mehta J Borges A. Extranodal follicular dendritic cell sarcoma-a review: “What the mind does not know the eye does not see”. Adv Anat Pathol 2021;28:21–9. 32991350 10.1097/PAP.0000000000000281

[7] Ge R Liu C Yin X. Clinicopathologic characteristics of inflammatory pseudotumor-like follicular dendritic cell sarcoma. Int J Clin Exp Pathol 2014;7:2421–9. 24966952 PMC4069939

[8] Chan JK Fletcher CD Nayler SJ Cooper K. Follicular dendritic cell sarcoma. Clinicopathologic analysis of 17 cases suggesting a malignant potential higher than currently recognized. Cancer 1997;79:294–313. 9010103

[9] Shia J Chen W Tang LH. Extranodal follicular dendritic cell sarcoma: clinical, pathologic, and histogenetic characteristics of an underrecognized disease entity. Virchows Arch 2006;449:148–58. 16758173 10.1007/s00428-006-0231-4

[10] Saygin C Uzunaslan D Ozguroglu M Senocak M Tuzuner N. Dendritic cell sarcoma: a pooled analysis including 462 cases with presentation of our case series. Crit Rev Oncol Hematol 2013;88:253–71. 23755890 10.1016/j.critrevonc.2013.05.006

[11] Cheuk W Chan JK Shek TW. Inflammatory pseudotumor-like follicular dendritic cell tumor: a distinctive low-grade malignant intra-abdominal neoplasm with consistent Epstein-Barr virus association. Am J Surg Pathol 2001;25:721–31. 11395549 10.1097/00000478-200106000-00003

[12] Deng S Gao J. Inflammatory pseudotumor-like follicular dendritic cell sarcoma: a rare presentation of a hepatic mass. Int J Clin Exp Pathol 2019;12:3149–55. 31934158 PMC6949706

[13] Swerdlow SH. International Agency for Research on Cancer & World Health Organization. WHO Classification of Tumours of Haematopoietic and Lymphoid Tissues. Revised 4th Lyon, France: International Agency for Research on Cancer; 2017.

[14] Selves J Meggetto F Brousset P. Inflammatory pseudotumor of the liver. Evidence for follicular dendritic reticulum cell proliferation associated with clonal Epstein-Barr virus. Am J Surg Pathol 1996;20:747–53. 8651355 10.1097/00000478-199606000-00013

[15] Yuan J Li XH LÜ YL Song X. Pathologic diagnosis of hepatic inflammatory pseudotumor and inflammatory pseudotumor-like follicular dendritic cell tumor. Linchuang Yu Shiyan Binglixue Zazhi 2007;23:39–42.

[16] Arber DA Kamel OW van de Rijn M. Frequent presence of the Epstein-Barr virus in inflammatory pseudotumor. Hum Pathol 1995;26:1093–8. 7557942 10.1016/0046-8177(95)90271-6

[17] Yu H Gibson JA Pinkus GS Hornick JL. Podoplanin (D2-40) is a novel marker for follicular dendritic cell tumors. Am J Clin Pathol 2007;128:776–82. 17951199 10.1309/7P8U659JBJCV6EEU

[18] Go H Jeon YK Huh J. Frequent detection of BRAF(V600E) mutations in histiocytic and dendritic cell neoplasms. Histopathology 2014;65:261–72. 24720374 10.1111/his.12416

[19] Pileri SA Grogan TM Harris NL. Tumours of histiocytes and accessory dendritic cells: an immunohistochemical approach to classification from the International Lymphoma Study Group based on 61 cases. Histopathology 2002;41:01–29. 10.1046/j.1365-2559.2002.01418.x 12121233

[20] Vermi W Giurisato E Lonardi S. Ligand-dependent activation of EGFR in follicular dendritic cells sarcoma is sustained by local production of cognate ligands. Clin Cancer Res 2013;19:5027–38. 23888072 10.1158/1078-0432.CCR-13-1275

[21] Gounder M Desai V Kuk D. Impact of surgery, radiation and systemic therapy on the outcomes of patients with dendritic cell and histiocytic sarcomas. Eur J Cancer 2015;51:2413–22. 26298731 10.1016/j.ejca.2015.06.109 PMC5087129

[22] Liu X Cao L Chin W Yu J Liu Y Zheng S. Epstein-Barr virus-negative inflammatory pseudotumor-like variant of follicular dendritic cell sarcoma of the liver: a case report and literature review. Clin Res Hepatol Gastroenterol 2021;45:101457. 32540141 10.1016/j.clinre.2020.05.007

